# First-line antiretroviral therapy durability in a 10-year cohort of naïve adults started on treatment in Uganda

**DOI:** 10.7448/IAS.19.1.20773

**Published:** 2016-06-17

**Authors:** Barbara Castelnuovo, Agnes Kiragga, Frank Mubiru, Andrew Kambugu, Moses Kamya, Steven J Reynolds

**Affiliations:** 1 Infectious Disease Institute, Makerere University, Kampala, Uganda; 2 School of Medicine, Makerere University, Kampala, Uganda; 3 Division of Intramural Research, National Institute of Allergy and Infectious Diseases, National Institutes of Health, Bethesda, MD, USA; 4 Johns Hopkins University School of Medicine, Baltimore, MD, USA

**Keywords:** antiretroviral treatment, treatments failure, long term outcomes

## Abstract

**Introduction:**

The majority of studies from resource-limited settings only report short-term virological outcomes of patients on antiretroviral treatment (ART). We aim to describe the long-term durability of first-line ART and identify factors associated with long-term virological outcomes.

**Methods:**

At the Infectious Diseases Institute in Kampala, Uganda, 559 adult patients starting ART in 2004 were enrolled into a research cohort and monitored with viral load (VL) testing every six months for 10 years. We report the proportion and cumulative probability of 1) achieving virologic suppression (at least one VL <400 copies/ml); 2) experiencing virologic failure in patients who achieved suppression (two consecutive VLs >1000 copies/ml or one VL >5000, for those without a subsequent one); 3) treatment failure (not attaining virologic suppression or experiencing virologic failure). We used Cox regression methods to determine the characteristics associated with treatment failure. We included gender, baseline age, WHO stage, body mass index, CD4 count, propensity score for initial ART regimen, VL, time-dependent CD4 count and adherence.

**Results:**

Of the 559 patients enrolled, 472 (84.8%) had at least one VL (67 died, 13 were lost to follow-up, 4 transferred, 2 had no VL available); 73.6% started on d4T/3TC/nevirapine and 26.4% on AZT/3TC/efavirenz. Patients in the two groups had similar characteristics, except for the higher proportion of patients in WHO Stage 3/4 and higher VL in the efavirenz-based group. Four hundred thirty-nine (93%) patients achieved virologic suppression with a cumulative probability of 0.94 (confidence interval (CI): 0.92–0.96); 74/439 (16.9%) experienced virologic failure with a cumulative probability of 0.18 (CI: 0.15–0.22). In the multivariate analysis, initial d4T/3TC/nevirapine regimen (hazard ratio (HR): 3.02; CI: 3.02 (1.66–5.44, *p<*0.001)) and baseline VL ≥5 log10 copies/ml (HR: 2.29; CI: 1.29–4.04) were associated with treatment failures; patients of older age (HR: 0.87 per five-year increase; CI: 0.77–0.99), with adherence >95% (HR: 0.04; CI: 0.02–0.11) and with higher time-dependent CD4 count (HR: 0.94 per 50 cells/µl increase; CI: 0.92–0.99, *p<*0.001) were less likely to experience treatment failure.

**Conclusions:**

The long-term virological outcomes from this cohort are promising and comparable to those from research-rich settings. Our results provide further evidence that efavirenz is associated with better virological outcomes.

## Introduction

Access to life-saving antiretroviral treatment (ART) has rapidly expanded in resource-limited settings over the past decade [1]. The greatest increase in the number of HIV-positive patients receiving ART was achieved in sub-Saharan Africa, where over 7.5 million people receive ART [2], resulting in a significant decline in HIV-related deaths as compared to the pre-ART period [3], by 22 to 29% [4].

In order to ensure sustainability of these improved survival outcomes, it is essential for HIV-infected individuals to achieve and maintain virologic suppression. Viral load (VL) testing is the gold standard for monitoring ART efficacy [5], but the majority of studies only report short-term virological outcomes on ART [6].

Partly due to the cost and complexity of VL testing, access to virologic monitoring remains limited in sub-Saharan Africa and only recently has routine VL monitoring being recommended by the WHO [7]. The vast majority of programmes have relied on immunological (CD4 cell count) monitoring to identify patients with ART treatment failure. Several reports from sub-Saharan Africa have demonstrated that these criteria are neither sensitive nor specific to identify patients with virologic failure and in need of second-line treatment [8, 9]. Programmes where patients are monitored through CD4 counts have reported very low rates of switches to second-line treatment, but this is unlikely to reflect the true durability of first-line treatment [10], since the majority of the patients with virologic failure may have remained unidentified [11–13].

In this study, we describe the durability and virological outcomes of first-line treatment in a cohort of Ugandan patients on ART monitored prospectively through biannual VL testing for 10 years, as well as the risk factors for first-line treatment failure.

## Methods

### Study site and population

The Infectious Diseases Institute (IDI) is an HIV Centre of Excellence [14] located in Mulago Teaching Hospital in Kampala with more than 30,000 patients enrolled in HIV care. Free ART has been provided by the Global Fund and the US President's Emergency Plan for AIDS Relief since April 2004. Patients are started on ART according to the current WHO and national guidelines after attending at least two counselling sessions. ART effectiveness is monitored at programme level through biannual CD4 counts; VL testing is available and it is performed *ad hoc* in patients with immunologic failure according to the WHO guidelines [7]; toxicity is also not routinely monitored but laboratory safety tests are performed at clinicians’ discretion. Laboratory testing is performed at the Makerere University–Johns Hopkins University Core Laboratory, which follows good laboratory practice guidelines and is certified by the College of American Pathologists.

Between April 2004 and April 2005, 559 consecutive patients starting ART were enrolled into a well-characterized cohort and followed up for 10 years.

### Study procedures

The study procedures have been described in detail elsewhere [15]. In summary, patients were evaluated by the study doctor and medication adherence counsellor at enrolment; during the follow-up they were evaluated by the study doctor and counsellor every three months, while they attended the general clinic for monthly ART prescription refill. ART was started according to the Ugandan and 2003 WHO guidelines [16, 17] in patients with WHO Stage 4 or having a CD4 count <200 cells/µl with stavudine (weight-adjusted), lamivudine and nevirapine (fixed-dose combination) or zidovudine, lamivudine (fixed-dose combination) and efavirenz.

At enrolment and follow-up, information about demographic characteristics, clinical and HIV history, vital signs, adherence and ART regimen was collected and a physical examination was performed. Adherence to ART was measured using multiple indicators: visual analogue scale, three- and seven-day recall and pill count. Reasons for non-adherence were also recorded. During the follow-up visits, adherence was assessed using the visual analogue scale, and ART toxicity and reason for ART substitution were recorded. Patients with two consecutive VLs >1000 copies/ml were considered eligible to be switched to a second-line regimen. Ritonavir-boosted lopinavir was the only protease inhibitor available up to 2013, when ritonavir-boosted atazanavir was made available at IDI.

Every six months, laboratory tests were performed, including complete blood cell count, liver and renal function tests, CD4 count by FACSCount (Becton Dickinson, San Jose, CA, USA) and, more recently, by FACSCalibur (Becton Dickinson), VL by Amplicor HIV-1 Monitor PCR Test version 1.5 (Roche Diagnostics, Indianapolis, IN, USA) and more recently, COBAS AmpliPrep/COBAS TaqMan HIV-1 Test version 2.0 (Roche Diagnostics, Indianapolis, IN, USA) and storage of 5 ml of plasma at –80°C for future testing.

The study was reviewed and approved by the Makerere University Faculty of Medicine Research and Ethics Committee (approval number 016-2004) and the Uganda National Council for Science and Technology (approval number MV 853).

Data were collected into an electronic medical record and periodically validated by a senior data entrant.

### Definitions

*Nevirapine-based ART regimens* refer to stavudine, lamivudine and nevirapine combinations whereas *efavirenz-based regimes* refer to zidovudine, lamivudine and efavirenz combinations.

*Virologic suppression* was defined as attaining at least one VL measurement <400 copies/ml after starting ART. For patients who achieved virologic suppression, *virologic failure* was defined as two consecutive VLs >1000 copies; this cutoff was chosen in accordance to the current WHO guidelines [7]. For those with one measurement above 1000 copies/ml and no following measurement available treatment failure was defined as VL >5000 copies/ml as per previous WHO guidelines [18]. *Treatment failure* was defined as either not attaining virologic suppression or experiencing virologic failure after suppression. *Time to treatment failure* was defined as either the time spent by patients who never achieved suppression with a VL >400 ml/copies or the time from ART start to the second VL >1000 or VL >5000 for those with no following measurement available.

### Statistical analysis

We used a chi-square test to compare categorical variables and Wilcoxon rank-sum test for continuous variables with non-normal distribution.

We reported the proportion of patients achieving virologic suppression and the cumulative probability stratified by gender and initial ART regimen. We described the proportion of patients with virologic failure while on first-line treatment and the cumulative probability of virologic failure stratified by gender and initial ART regimen. The cumulative probabilities were estimated using Kaplan–Meier curves and all differences in survival probabilities were compared using the log-rank test.

We used propensity score methods to predict the probability of regimen allocation at ART initiation. We used Cox regression methods to determine the factors associated with treatment failure. We included gender, baseline age, body mass index, CD4 count, VL (<5 log10 copies/ml and ≥5 log10 copies/ml), WHO staging (Stage 1/2 and Stage 3/4), the propensity score for initial ART regimen allocation, time-dependent CD4 count measurement and time-dependent adherence levels stratified by levels above and equal to or below 95%, as studies have demonstrated that these levels are necessary to obtained sustained suppression [19, 20]. Predictors with *p*≤0.2 in the unadjusted analysis and those of clinical significance were included in the multivariable analysis. The analysis was performed using STATA^®^ version 12.2 (StataCorp, College Station, TX, USA).

## Results

Of the 559 patients enrolled in the study, 472 (84%) were included in this analysis; 85 patients were excluded because they did not reach six months of follow-up (67 died, 13 were lost to follow-up, 4 transferred, 1 withdrew consent) [21] and two because they did not have a VL measurement after the baseline visit.

Characteristics of the patients included in the analysis by the initial ART regimen (nevirapine-based *versus* efavirenz-based) are shown in Table 1. Patients in the two groups had similar characteristics, except for the higher proportion of patients in WHO Stages 3 and 4 as opposed to Stages 1 and 2 in the nevirapine-based group as compared to the efavirenz-based group (*p*<0.001). In addition, there were higher VL measurements in the efavirenz-based group as compared to the nevirapine-based group (*p*=0.019).

**Table 1:** Patient characteristics by initial antiretroviral treatment regimen

Characteristics	Nevirapine^a^ *N=*349 (74%)	Efavirenz-based^a^ *N=*123 (26%)	*p*
Gender, female	245 (70.2%)	80 (65.0%)	0.288
Age in years, median (IQR)	34 (30–42)	35 (31–41)	0.504
WHO Stages 3 and 4	322 (92.3%)	97 (78.9%)	0.000
BMI (Kg/m^2^), median (IQR)	20.3 (18.4–22.6)	19.7 (18.0–21.6)	0.079
Hb g/dL, median (IQR)	11.8 (10.5–13.0)	11.7 (10.5–13.2)	0.683
CD4 count in cells/µl, median (IQR)	101 (30–170)	103 (33–159)	0.912
HIV RNA log copies/ml, median (IQR)	5.4 (5.1–5.7)	5.6 (5.2–5.8)	0.019

a Nevirapine-based (nevirapine+stavudine+lamivudine); efavirenz-based (efavirenz+zidovudine+lamivudine). WHO, World Health Organization; BMI, body mass index.

### Virologic suppression and virologic failure

**Figure 1:**
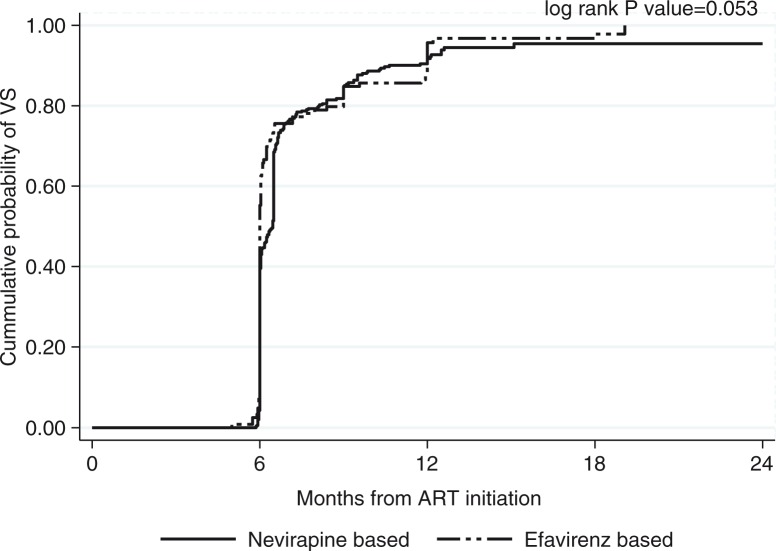
Cumulative probability of virologic suppression in 472 patients enrolled in the cohort and who reached six months of follow-up by initial antiretroviral treatment regimen (nevirapine-based versus efavirenz-based).

Of the 472 patients, 33 (7%) never achieved virologic suppression during the first 24 months, of which 5 (15%) died, 6 (18%) were lost to follow-up, 2 (6%) withdrew consent and 20 (61%) were switched to a second-line regimen. The cumulative probability of attaining virologic suppression was 0.94 (95% confidence interval (CI): 0.92–0.96) (Figure 1). The cumulative probability of attaining virologic suppression in patients started on a nevirapine-based regimen was lower compared to patients started on efavirenz-based regimens with a borderline statistical significance (*p*=0.053).

**Figure 2:**
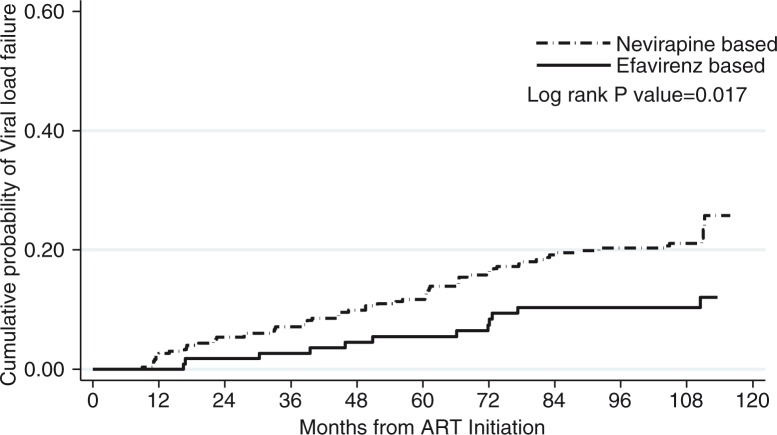
Cumulative probability of virologic failure in the 439 patients who achieved virologic suppression by initial antiretroviral treatment regimen (nevirapine-based versus efavirenz-based).

Over the 10-year follow-up period, 74/439 (17%) patients who achieved suppression experienced virologic failure (two consecutive VLs >1000 copies/ml or one VL >5000 copies/ml, if no subsequent VL measurement was available) after a median time of 51 months (95% CI: 28–72), of which 7/74 (9.5%) died. The cumulative probability of experiencing virologic failure was higher in patients started on nevirapine (0.22; CI: 0.17–0.26) as compared to patients started on efavirenz (0.10; CI: 0.06–0.18, *p=*0.0017) (Figure 2).

### Treatment failure

**Figure 3:**
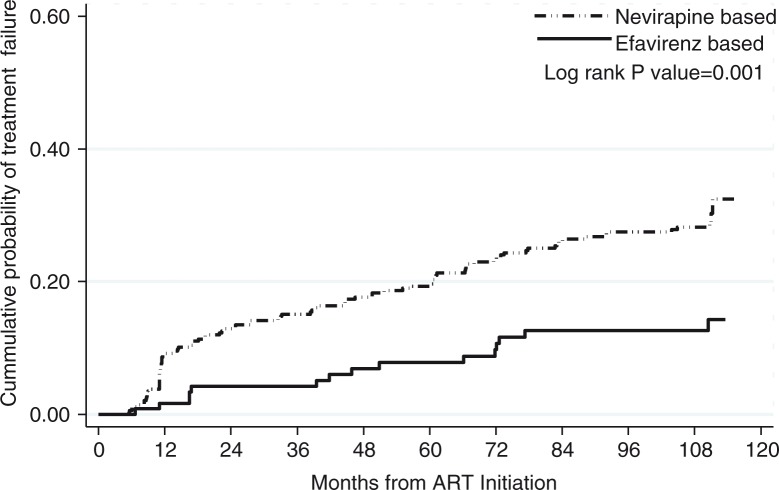
Cumulative probability of treatment failure by initial antiretroviral treatment regimen.

A total of 107/472 (22.7%) patients experienced treatment failure, of whom 33 never attained virologic suppression and 74 experienced virologic failure after achieving virologic suppression with a median (IQR) time to treatment failure of 33 (IQR: 11.2–67) months. The cumulative probability of experiencing treatment failure was higher in patients started on nevirapine-based regimens (0.28; 95% CI: 0.24–0.34) as compared to patients started on efavirenz-based regimens (0.13; 95% CI: 0.08–0.20, *p*=0.001) (Figure 3). Of note, while the probability of treatment failure increased steadily in the patients started on nevirapine-based regimens, the cumulative probability of failing (0.13; 95% CI: 0.08–0.20) did not further increase after seven years on ART in patients started on efavirenz-based regimens, with no patients experiencing treatment failure in the eight- to ten-year follow-up period (Figure 3).

In the multivariate analysis, patients started on nevirapine-based regimens were almost three times more likely to experience treatment failure (hazard ratio (HR): 3.02; 95% CI: 1.66–5.44); baseline VL ≥5 log10 copies/ml was also associated with treatment failure (HR: 2.29, CI: 1.29–4.04). Older age (HR: 0.87 per five-year increase; CI: 0.77–0.99), adherence >95% as compared to those with ≤95% adherence (HR: 0.04; CI: 0.02–0.11) and 50 cells/µl increase in time-dependent CD4 count (HR: 0.94; CI: 0.94–0.99) were protective for treatment failure (Table 2).

**Table 2:** Cox regression analysis of factors associated with treatment failure

Baseline characteristic	Crude HR (95% CI)	*p*	Adjusted HR (95% CI)	*p*
Gender				
Female	1.00			
Male	0.95 (0.63–1.45)	0.930		
Age per 5-year increase	0.88 (0.78–1.00)	0.052	0.87 (0.77–0.99)	0.034
Viral load				
<5 log10 copies/ml	1.00		1.00	
≥ 5 log10 copies/ml	1.51 (0.89–2.57)	0.128	2.29 (1.29–4.04)	0.004
WHO stage				
1 and 2	1.00		1.00	
3 and 4	2.63 (1.07–6.47)	0.035	0.84 (0.28–2.48)	0.759
CD4 count per 50 cells/µl increase	0.94 (0.84–1.05)	0.26		
Baseline BMI	0.99 (0.945–1.04)	0.822		
ART regimen				
Efavirenz-based^a^	1.00		1.00	
Evirapine-based^a^	2.41 (1.40–4.16)	0.002	3.02 (1.66–5.44)	<0.001
Time-varying characteristics				
Mean adherence^b^ (%)				
≤ 95%			1.00	
> 95%	0.11 (0.05–0.24)	<0.001	0.04 (0.02–0.11)	<0.001
CD4 count per 50 cells/µl increase	0.71 (0.65–0.77)	<0.001	0.94 (0.92–0.99)	<0.001

a Nevirapine-based (nevirapine+stavudine+lamivudine); efavirenz-based (efavirenz+zidovudine+lamivudine);

b measured by visual analogue scale. CI, confidence interval; HR, hazard ratio; WHO, World Health Organization; ART, antiretroviral treatment; BMI, body mass index.

## Discussion

To our knowledge this is the first study from sub-Saharan Africa to report virological outcomes in an African cohort of patients on ART for 10 years.

The ultimate goal of ART is the greatest possible reduction in VL for as long as possible. Achieving and maintaining virologic suppression is associated with reduction in mortality and morbidity [22, 23]; in addition, undetectable levels of viremia contribute to prevent the accumulation of resistance mutations [24]. Virologic success is also the foundation of the strategy of using ART as treatment as prevention [25].

The WHO global strategy for prevention and assessment of HIV drug resistance set up an ideal target of 70% of virologic suppression by year 1 on ART [26]. In our cohort of 559 patients started on ART, 72% (data not shown) achieved VL <400 copies/ml during the first year; of those with at least six months of follow-up, only 7% never attained virologic suppression while on first-line ART. We believe this is an impressive accomplishment compared to the set target, as well as to other programmes in sub-Saharan Africa, where overall 82% of patients achieved virologic suppression by year 1 on ART [6]. Our results are also encouraging in view of the 90-90-90 target set by UNAIDS.

In addition, after 10 years on ART only 17% of patients experienced virologic failure while on first-line treatment; the risk of virologic failure continued to steadily increase up to seven years of follow-up and levelled off afterwards, with only 7% (5/74) of patients experiencing virologic failure after the seventh year of follow-up. Although it has been noted that duration of treatment is associated with decreased adherence [27], a phenomenon also known as “treatment fatigue” [28], the duration of virologic suppression has been reported to be inversely correlated to virologic failure [29]. This finding is in agreement with the overall declining incidence of virologic failure observed in our cohort. One possible explanation for this decrease in risk of virologic failure over time is that, in those who attain initial suppression, lower levels of drug exposure are required to maintain suppression and prevent subsequent failure due to very low levels of residual viremia [30]. As a consequence of this, ART doses missed after long-term virologic suppression may have fewer negative consequences as compared to doses missed before or shortly after achieving virologic suppression.

Another important finding of our analysis is that efavirenz is associated with superior virological outcomes as compared to nevirapine. In this cohort, patients who were started on nevirapine-based regimens had three times higher risk of treatment failure. Interestingly, the difference in risk of treatment failure emerged in the first two years and levelled off afterwards (Figure 3). A recent analysis of cohorts from the United States and Europe also found an increased risk in treatment failure in patients started on nevirapine as compared to efavirenz-based regimens [31]. In addition, a recent large analysis of a South African cohort showed that patients started on nevirapine were 80% more likely to experience failure as compared to those started on efavirenz, regardless of the nucleoside backbone drug used [32], while in a recent cross-sectional study from Uganda, use of efavirenz was associated with a 50% decrease in risk of treatment failure [33]. We hypothesize that although nevirapine performance in attaining initial virologic suppression and short-term sustainment of virologic suppression seems to be inferior as compared to efavirenz, this effect fades away over time once suppression is achieved and maintained.

Despite published evidence suggesting the superiority of efavirenz as compared to nevirapine, a limitation of this study is that at the time of starting patients on ART, the prescribed antiretroviral drugs were formulated as a fixed-dose combination. In particular, nevirapine-based regimens contain stavudine, a drug known to cause several side effects [34–36], which in turn could have a negative impact on adherence. It is therefore not possible to be conclusive on the contribution to treatment failure of each drug contained in the regimens.

## Conclusions

This is the first study to describe long-term first-line ART durability in our setting. As access to ART expands, and as prospective monitoring has been introduced in many countries in sub-Saharan Africa, including Uganda [37], we believe that our findings are important to predict the rate of treatment failure and to project the amount of second-line drugs needed. In addition, this study provides further evidence that efavirenz is associated with better virological outcomes and should be considered the NNRTI of choice in patients starting ART in our setting.
